# Time-Series Analysis of Atmospheric Pollution and *Mycoplasma pneumoniae* Infections in Children

**DOI:** 10.1155/cjid/8860382

**Published:** 2025-09-24

**Authors:** Haiyan Zhang, Meimei Lai, Hao Dong, Luoman Yan, Lei Zhang

**Affiliations:** ^1^Chengdu Women's and Children's Central Hospital, School of Medicine, University of Electronic Science and Technology of China, Chengdu 611731, China; ^2^The Department of Pediatrics, The Affiliated Hospital of Southwest Medical University, Sichuan Clinical Research Center for Birth Defects, Luzhou 646000, Sichuan, China

**Keywords:** air pollution, children, MP infection, time-series

## Abstract

**Objective:** Aimed to analyze the relationship between air pollution and *Mycoplasma pneumoniae* (MP) infection in children in Chengdu.

**Method:** Data on outpatient and inpatient cases of MP infection among children at Chengdu Women and Children's Central Hospital from 2019 to 2023 were retrospectively collected. Air pollution and meteorological data from the same period were also obtained. A generalized additive model (GAM) was established using R statistical software to examine the impact of different air pollutant concentrations on MP infection incidence in children. The relationship between pollutant concentrations and MP infection rates was further analyzed by stratifying data by age, sex, and season.

**Results:** From 2019 to 2023, a total of 21,075 outpatient and emergency cases and 6964 inpatient cases of MP infection were reported among children at Chengdu Women and Children's Central Hospital. A 10-μg/m^3^ increase in the daily concentration of particulate pollutants (PM_2.5_, PM_10_) had the most significant delayed effect on outpatient MP infection incidence at a 6-day lag (lag 06), although the cumulative lag effect was not statistically significant. When the average daily concentration of gaseous pollutants (SO_2_) increased by 10 μg/m^3^, the strongest lag effects on outpatient and inpatient MP infections were observed at 7-day lags (lag 07 and lag 7, respectively). In the single air pollutant model, age-stratified analysis showed that SO_2_ concentration had the most significant correlation with the incidence of outpatient and inpatient MP infections in children under 6 years of age, while nitrogen dioxide (NO_2_) concentration had the most significant correlation in children over six. Sex-stratified analysis indicated that SO_2_ levels were most significantly associated with MP infection in males, whereas NO_2_ were most strongly correlated in females. Among outpatients, SO_2_ had the most substantial effect on MP infection incidence across sexes. Seasonal stratification revealed that the impact of air pollution on MP infection was greater in autumn and winter than in spring and summer.

**Conclusion:** Increased air pollution levels in Chengdu from 2019 to 2023 had a measurable impact on MP infection incidence in both inpatient and outpatient children, with notable lag and cumulative lag effects. These effects were more pronounced in autumn and winter, highlighting the need for targeted early warning systems to monitor air pollutant concentrations. Such efforts could play a crucial role in protecting vulnerable populations and reducing MP infection risks in children.

## 1. Introduction

The International Organization for Standardization (ISO) defines air pollution (also known as atmospheric pollution) as the phenomenon in which certain substances are introduced into the atmosphere by human activities or natural processes, attaining sufficient concentration and persisting for sufficient duration to endanger human comfort, health, well-being, or the environment. Air pollution poses a significant threat to human health and is linked to various adverse health effects. Common air pollutants include suspended particulate matter, gaseous pollutants, and volatile organic compounds, such as NO, NH_3_, O_3_, SO_2_, and CO [1]. With accelerating urbanization, air pollution has become increasingly prominent, not only exacerbating chronic cardiovascular diseases but also increasing susceptibility to respiratory infections [2], primarily through two mechanisms. First, in the oxidative stress pathway, exposure to air pollutants triggers oxidative stress, generating excessive free radicals and reactive oxygen species (ROS) [3–5]. This damages cellular and tissue structures in the respiratory system, impairing defenses against bacterial/viral infections and facilitating pathogen invasion (e.g., *Mycoplasma pneumoniae* [MP]). Second, in the microbiota dysbiosis pathway [6], air pollutants disrupt respiratory microbiota homeostasis, particularly reducing commensal α-hemolytic streptococci (*Streptococcus* spp.) and consequently promoting proliferation of pathogenic flora (e.g., *Mycoplasma*) and elevating infection risks. Mycoplasma, a prokaryotic microorganism lacking a cell wall, falls between bacteria and viruses taxonomic classification. It is the smallest known microorganism capable of independent survival, with a diameter of 50–300 nm and a simple structure [7]. Following the global introduction of the *Streptococcus pneumoniae* vaccine, MP has become the leading cause of community-acquired pneumonia (CAP) in children, accounting for 10%–40% of CAP cases and 4%–8% of MP cases in Europe. MP is highly transmissible through droplets or direct contact, especially in densely populated, enclosed, or poorly ventilated environments. The incubation period ranges from 2 to 4 weeks, with epidemics occurring every 3–7 years [1, 7, 8]. Although MP infections are typically self-limiting, they can lead to refractory pneumonia and nonrespiratory complications, such as myocarditis, arthritis, and thrombosis. In severe cases, untreated infections may result in serious complications or even death [9, 10]. Therefore, studying the correlation between air pollution and MP is imperative.

Previous studies have predominantly focused on establishing correlations between air pollutants and broad-spectrum respiratory diseases, while seldom exploring pathogen-specific pathogenesis linked to particular etiological agents. Notably, there exists a significant knowledge gap regarding the interaction mechanisms of pollutants and cyclically emerging pathogens, such as MP. Given the heightened susceptibility of children—due to their developing respiratory systems and greater exposure to air pollutants—studying the correlation between air pollution and MP infection is essential [11–15]. Time-series analysis is a widely used method for assessing the effects of air pollution on public health. In this study, we applied the time-series analysis and a generalized additive model (GAM) to investigate the relationship between air pollutant concentrations and MP infection in children in Chengdu. We further examined the effects of age, sex, and seasonal variations to identify high-risk groups and develop early warning strategies, providing a theoretical foundation for MP prevention and treatment.

## 2. Data and Methods

### 2.1. Data Sources

This study retrospectively collected data on outpatient, emergency, and inpatient cases of MP infection at Chengdu Women and Children's Central Hospital from 2019 to 2023. The dataset included patient visit times, sex, age, and diagnosis codes based on the International Classification of Diseases (ICD), using the disease code A49.300 as the research sample. Meteorological data (average daily temperature and relative humidity) and atmospheric pollutants (PM_10_, PM_2.5_, SO_2_, NO_2_, CO, O_3_) for the same period were obtained from the Chengdu Meteorological Bureau (http://sc.cma.gov.cn/ds/cd/).

### 2.2. Methods

A GAM was used to investigate the relationship between air pollution and MP infections in children. GAM is suitable not only for analyzing a variety of complex nonlinear relationships but also for correcting long-term and seasonal trends in disease outcomes, meteorological factors, and other confounders. Since MP infection cases represent a relatively small proportion of the total pediatric population in Chengdu, they were assumed to follow a Poisson distribution. The Poisson-based GAM model was fitted using the R statistical software package. To minimize confounding effects–such as long-term trends, seasonal patterns, day-of-week variations, and holiday influences–various pollutants were introduced into the GAM as linear variables. The model was then used to assess the impact of different air pollutants on MP infection incidence in children.

The model is defined as follows:

(1) $$\begin{array}{c} Y_{t}\;∼\text{ Poisson }\left({µ}_{t}\right), \end{array}$$

(2) $$\begin{array}{c} \mathrm{Log}\;\left(t\right)=\beta X_{t}+s\left(t,dft\right)+s\left(Zt,\text{dft}\right)+\text{Holiday}+\text{DOW}+ɑ, \end{array}$$

where *t* represents the observation date, *Y*_*t*_ indicates the number of people who became ill due to MP infection on day *t*, and μt represents the expected number of infections on day *t*. *X*_*t*_ indicates the concentration of atmospheric pollutants (including SO_2_, O_3_, CO, PM_10_, NO_2_, and PM_2.5_) on the day *t*, β is the regression coefficient, *s* is the natural smooth spline function, *Zt* represents the meteorological factors (including the average temperature and relative humidity) of day *t*, and dft is the degree of freedom (DOF) of the nonparametric smoothing function. The holiday variable is a binary indicator representing Chinese legal holidays; DOW indicates the day of the week (values range from 1 (Sunday) to 7 (Saturday)), and ɑ is the intercept. To adjust for the delay and nonlinear confounding effects of temperature and humidity, we made several adjustments to the DOFs in the model and, combined with previous studies, adopted a distributed hysteresis nonlinear model with three DOFs in the natural smoothing spline function. According to the partial autocorrelation function (PACF), the DOF for the summary nonparametric smoothing function was determined using 5 years to control seasonal and long-term trends in the time-series dataset.

In the single-lag effect study, lag 0–lag 07 were selected for analysis. Here, lag 0 represents pollutant concentration on the same day, lag 01 represents pollutant concentration on the previous day, and so forth. This approach helps determine the most significant lag time for each air pollutant in the MP infection model for children. In the cumulative lag effect study, lag 1–lag 7 were selected for analysis. Lag 1 represents the average concentration of pollutants on the current day and the previous day, lag 2 represents the average concentration of pollutants on the current day and the two preceding days, and so on. This method was used to determine the maximum cumulative lag time for each air pollutant in relation to MP infection in children. Other pollutants were included in the nitrogen pollution model, and the stability of the model was evaluated using a multipollutant model. At the same time, the data were analyzed by gender, age, and season to analyze the relationship between the concentration of air pollutants in different subgroups and MP infection in children.

The study examined the following indicators: (1) air pollutant concentrations and meteorological parameters in Chengdu from 2019 to 2023; (2) correlation analysis of air pollutant concentrations in Chengdu from 2019 to 2023; (3) the number of children infected with MP in Chengdu Women and Children's Central Hospital from 2019 to 2023; and (4) effects of single air pollutants on MP infection in children. (5) Effects of air pollutants on MP infection in children were analyzed by stratifying them by sex, age, and season.

### 2.3. Statistical Processing

Statistical analysis was conducted using SPSS (Version 22.0; IBM Corp., Armonk, NY, USA). Descriptive statistics of weather data and air pollutant concentrations included the mean value, standard deviation, minimum value, maximum value, and percentile (P5, P25, P50, P75, and P95). Pearson's rank correlation was used for correlation analysis. The generalized additive Poisson regression model was used for the time-series analysis using the dlnm software package in R software. The results are presented as the excess risk (ER) and 95% confidence interval (CI) for the daily rate of increase in childhood MP infection per 10 μg/m^3^ increase in air pollutant concentrations.

## 3. Results

### 3.1. Atmospheric Pollutant Concentrations and Meteorological Parameters

**Figure 1:**
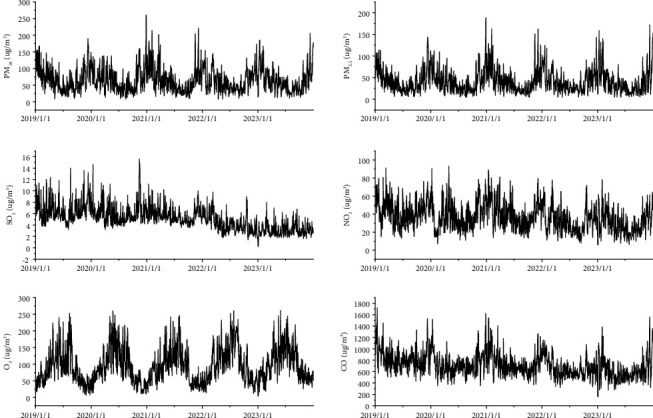
Time-series chart of air pollutant concentrations in Chengdu from 2019 to 2023.

A general description of atmospheric pollutant concentrations and meteorological parameters in Chengdu from 2019 to 2023 is presented in Table 1. Based on the concentration limit of ambient air pollutants in the National Ambient Air Quality Standard (GB3095-2013), the average concentration of PM_2.5_ was higher than the national secondary standard. Seasons were divided into spring, summer, autumn, and winter. The concentrations of all air pollutants fluctuated widely. Except for O_3_, the concentrations of the other air pollutants peaked in winter, as shown in Figure 1.

### 3.2. Incidence of MP Infection in Children, Concentration of Air Pollutants in Chengdu, and Correlation Analysis of Meteorological Factors

The incidence of MP infection in children in Chengdu from 2019 to 2023, concentration of air pollutants, and correlation analysis of meteorological factors are shown in Table 2. The Pearson rank correlation analysis showed that PM_2.5_, PM_10_, CO, SO_2_, O_3_, and NO_2_ were all correlated (all *p* < 0.05), and PM_2.5_ was highly positively correlated with PM_10_ (*r* = 0.953, *p* < 0.01). O_3_ was negatively correlated with the other air pollutants (*p* < 0.05). Among the meteorological factors, average daily temperature was positively correlated with O_3_ (*p* < 0.01) and negatively correlated with other atmospheric pollutants (*p* < 0.01). Relative humidity was positively correlated with CO (*p* < 0.01) and negatively correlated with other air pollutants (*p* < 0.01). The incidence of MP infection in children was negatively correlated with the concentration of atmospheric pollutants (PM_2.5_, PM_10_, CO, SO_2_, and NO_2_) (*p* < 0.01), but not with atmospheric pollutants (O_3_) or meteorological factors (daily average temperature and relative humidity) (Table 2).

### 3.3. Outpatient and Inpatient Data of MP Infection in Children

From 2019 to 2023, the total number of outpatients and emergency children with MP infection in Chengdu Women and Children's Central Hospital was 21,075 (male: 9783, female: 11,292) (Table 3). The total number of inpatients was 6964 (male: 3475, female: 3489) (Table 4). The leading group of children with MP infection was children under 6 years of age. The number of outpatient visits and hospitalizations in autumn and winter was higher than those in spring and summer.

### 3.4. Maximum Lag Time of the Model Results for the Impact of a Single Air Pollutant on MP Infection in Children and Its Health Effects

**Figure 2:**
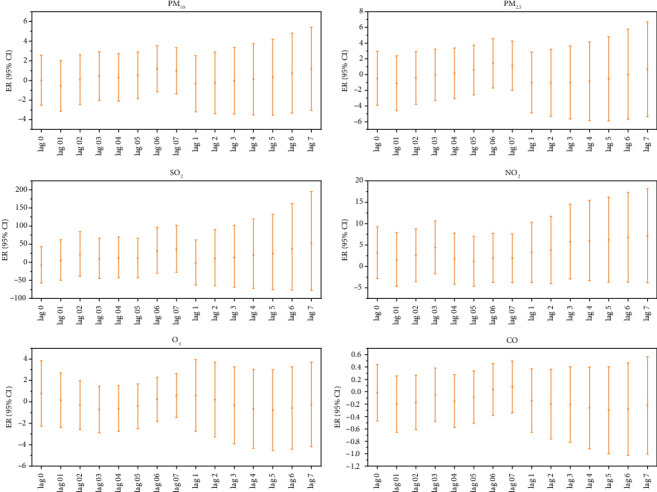
Lag effect and cumulative lag effect diagrams of outpatient children's MP infection for each 10 μg/m^3^ increase in air pollutant concentrations. Note: Log 01–07 represents the lag effect, while Log 1–7 represents the cumulative lag effect.

The analysis of a single air pollutant model showed that every 10 μg/m^3^ increase in the concentration of gaseous pollutants (SO_2_, NO_2_, CO) had a lag effect and a cumulative lag effect on the incidence of MP infection in outpatient children, with a lag of 7, 3, and 1 days, respectively. The cumulative lag effects at 7 and 5 days were the most significant. The maximum cumulative lag effect of SO_2_ and NO_2_ is consistent and is most significant when the cumulative lag is 7 days; the lag effect and cumulative lag effect on the incidence of MP infection in outpatient children are consistent when the concentration of particulate pollutants (PM_2.5_ and PM_10_) increases by 10 μg/m^3^, and the lag effect is the largest when the lag is 6 days. However, the cumulative lag effect is not statistically significant (Figure 2).

**Figure 3:**
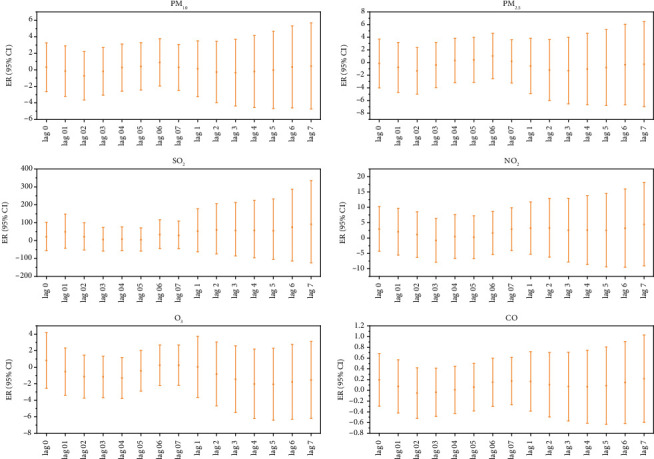
Lag effect and cumulative lag effect diagrams of hospitalized children's MP infection for each 10 μg/m^3^ increase in air pollutant concentrations. Note: Log 01–07 represents the lag effect, while Log 1–7 represents the cumulative lag effect.

Each 10 μg/m^3^ increase in the concentration of gaseous pollutants (SO_2_, O_3_) had a lag effect and a cumulative lag effect on the incidence of MP infection in hospitalized children. The effect was most significant when the lag was 1 or 3 days, and the cumulative lag was 7 or 5 days. The lag and cumulative lag effects of particulate pollutants (PM_2.5_, PM_10_) and other gaseous pollutants (NO_2_ and CO) were not statistically significant (Figure 3).

### 3.5. Effects of Air Pollutants on MP Infection in Children Stratified by Age, Sex, and Season

**Figure 4:**
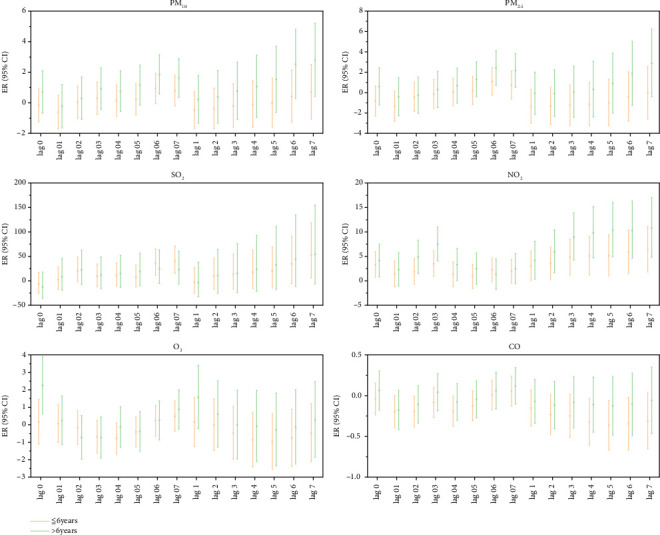
Lag effect and cumulative lag effect diagrams of outpatient children's MP infection by age group for each 10 μg/m^3^ increase in air pollutant concentrations. Note: Log 01–07 represents the lag effect, while Log 1–7 represents the cumulative lag effect.

Age stratification analysis showed that every 10 μg/m^3^ increase in the concentration of gaseous pollutants SO_2_, NO_2_, and CO had statistical significance on the lag and cumulative lag effects on the incidence of MP infection in outpatient children under 6 years of age (*p* < 0.05). Increased SO_2_ concentration had the most significant impact on the incidence of MP infections in outpatient children. The ER value corresponding to the lag effect was 40.59, and the ER value corresponding to the cumulative lag effect was 52.28. Every 10 μg/m^3^ increase in the concentration of particulate pollutants PM_10_, PM_2.5_, NO_2_, and O_3_ had a statistically significant lag effect on the incidence of MP infection in outpatient children over 6 years of age. An increase in the NO_2_ concentration had the most significant impact on the incidence of MP infection in outpatient children, and the ER value corresponding to the lag effect was 7.48. The cumulative lag effect corresponded to an ER value of 10.77 (Figure 4).

**Figure 5:**
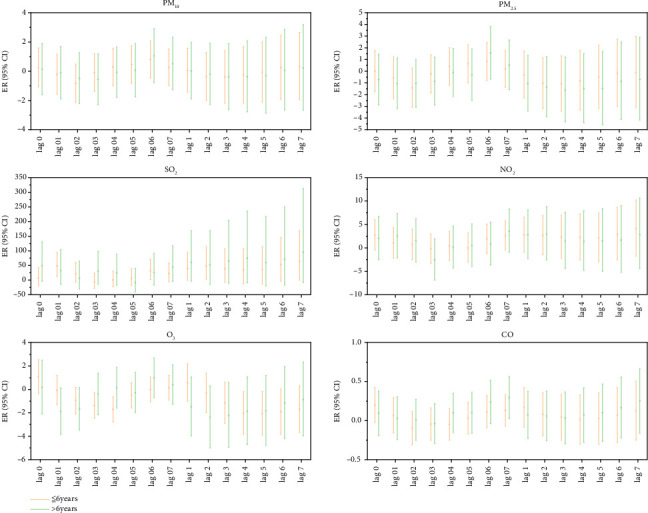
Lag effect and cumulative lag effect diagrams of hospitalized children's MP infection by age group for each 10 μg/m^3^ increase in air pollutant concentrations. Note: Log 01–07 represents the lag effect, while Log 1–7 represents the cumulative lag effect.

Each 10 μg/m^3^ increase in the concentration of gaseous pollutants SO_2_ and O_3_ had statistical significance on the lag effect and cumulative lag effect of MP infection in hospitalized children under 6 years of age (*p* < 0.05). The increase in SO_2_ concentration had the most significant impact on the incidence of MP infection in hospitalized children, and the ER value corresponding to the lag effect was 47.85, respectively. The ER values corresponding to cumulative lag effect were 48.45. The lag effect on MP infection in hospitalized children over 6 years of age was statistically significant for every 10 μg/m^3^ increase in gaseous pollutant CO concentration (*p* < 0.05), and the ER value was 0.29. There was no statistically significant lag effect or cumulative lag effect on MP infection in hospitalized children of different ages with increased PM_10_, PM_2.5_, and gaseous pollutant (NO_2_) (all *p* > 0.05) (Figure 5).

**Figure 6:**
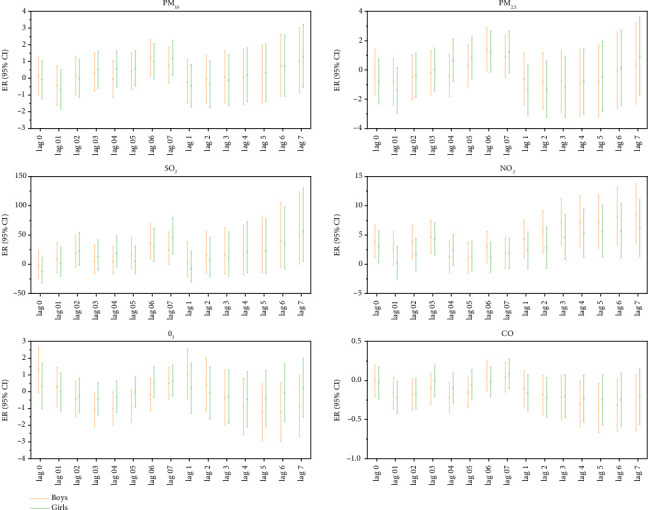
Lag effect and cumulative lag effect diagrams of outpatient children's MP infection by gender for each 10 μg/m^3^ increase in air pollutant concentrations. Note: Log 01–07 represents the lag effect, while Log 1–7 represents the cumulative lag effect.

Gender-stratified analysis showed that every 10 μg/m^3^ increase in the concentration of particulate pollutants (PM_10_) and gaseous pollutants (SO_2_, NO_2_, CO) had the same lag effect and cumulative lag effect on outpatient children of different genders. The increase in SO_2_ concentration had the most significant impact on the incidence of MP infections in outpatient children of both sexes. The ER values corresponding to the lag effect and cumulative lag effect were male: 35.98, 49.90, and female: 45.32, 55.53. The increase in the concentration of gaseous pollutants (O3) only had a lag effect on the incidence of MP infection in male outpatient children; the corresponding ER value was −1.07, and the cumulative lag effect was not statistically significant (Figure 6).

**Figure 7:**
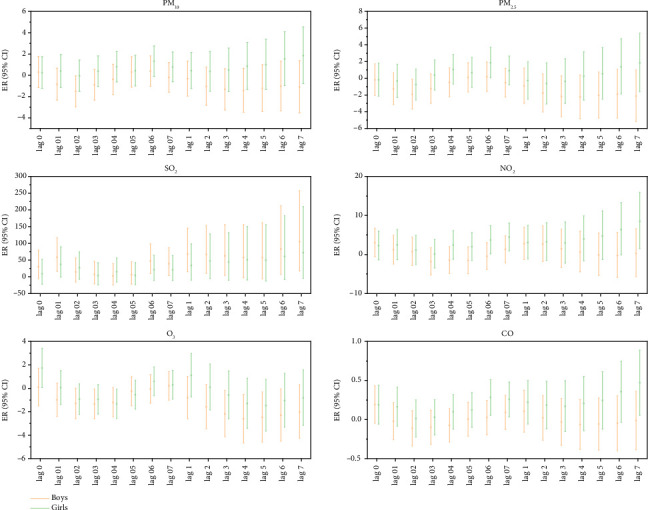
Lag effect and cumulative lag effect diagrams of hospitalized children's MP infection by gender for each 10 μg/m^3^ increase in air pollutant concentrations. Note: Log 01–07 represents the lag effect, while Log 1–7 represents the cumulative lag effect.

Particulate pollutants (PM_10_, PM_2.5_) and gaseous pollutants (SO_2_, O_3_) with an increase of 10 μg/m^3^ had a statistically significant lag effect on the incidence of MP infection in male hospitalized children (*p* < 0.05). Increased SO_2_ concentration had the most significant effect on the incidence of MP infection in hospitalized male children. The ER values corresponding to the lag and cumulative lag effects are 58.62 and 105.12, respectively. Each 10 μg/m^3^ increase in the concentration of gaseous pollutants (NO_2_ and CO) had a statistically significant and cumulative lag effect on the incidence of MP infection in female hospitalized children. The increase in NO_2_ concentration had the most significant impact on the incidence of MP infection in hospitalized female children, and the ER values corresponding to the lag and cumulative lag effect were 4.44 and 8.48, respectively, Figure 7.

**Figure 8:**
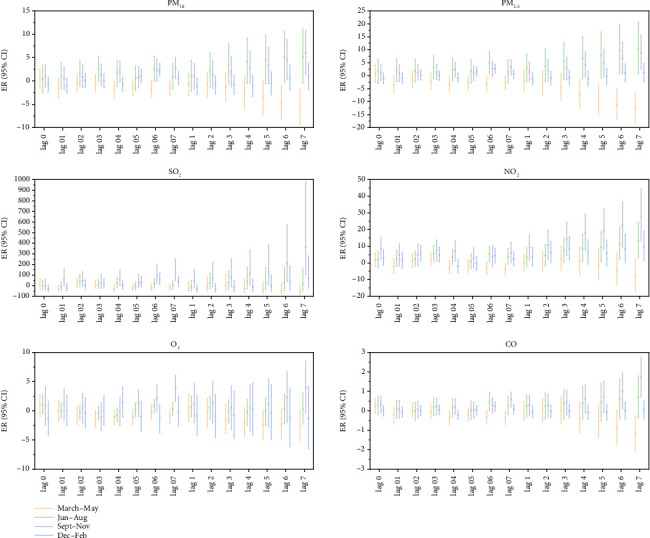
Lag effect and cumulative lag effect diagrams of outpatient children's MP infection by season for each 10 μg/m^3^ increase in air pollutant concentrations. Note: Log 01–07 represents the lag effect, while Log 1–7 represents the cumulative lag effect.

Seasonal stratification analysis showed that every increase in PM_10_, PM_2.5_, and NO_2_ concentrations by 10 μg/m^3^ had statistically significant lag effects on MP infection in outpatient children in the other three seasons except summer, and PM_2.5_ had a more significant impact in autumn, with an ER value of 5.48. NO_2_ had a more significant effect in winter, with an ER value of 13.91. When the concentration of SO_2_, CO, and O_3_ increased by 10 μg/m^3^, the lag effect on MP infection in outpatient children was statistically significant only in autumn, among which the lag effect and cumulative lag effect were both the largest, and the ER value corresponding to the lag effect was 160.00. The ER corresponding to the cumulative lag effect was 651.15 (Figure 8).

Each increase in SO_2_ and O_3_ concentrations by 10 μg/m^3^ had a statistically significant lag effect on MP infection in hospitalized children only in spring, in which SO_2_ had the most significant lag effect and cumulative lag effect, and the corresponding ER value was −70.94, respectively.

**Figure 9:**
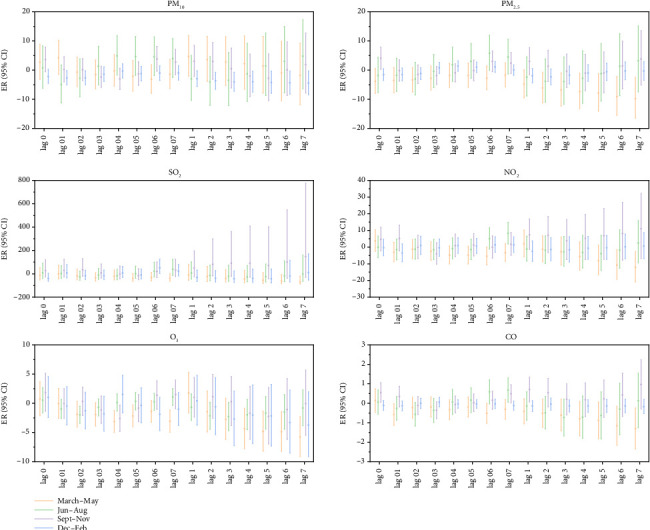
Lag effect and cumulative lag effect diagrams of hospitalized children's MP infection by season for each 10 μg/m^3^ increase in air pollutant concentrations. Note: Log 01–07 represents the lag effect, while Log 1–7 represents the cumulative lag effect.

When the concentration of PM_10_, PM_2.5_, and NO_2_ increased by 10 μg/m^3^, the lag effect and cumulative lag effect of MP infection in hospitalized children were statistically significant only in winter, among which NO_2_ had the most considerable lag effect and cumulative lag effect, and the ER value corresponding to the lag effect was −8.53. The cumulative lag effect corresponded to an ER value of −11.57 (Figure 9).

## 4. Discussion

This study is one of the few to examine the relationship between air pollutant exposure and the onset of MP infections in children, particularly in a densely populated city (Chengdu, China). A previous study in Thailand indicated that SO_2_ and PM_2_._5_ negatively impact the respiratory system and exacerbate respiratory issues. This study's model noted that particulate pollutants trigger inflammation and cellular damage through free radicals and ROS [16]; excessive inflammation and respiratory damage increase susceptibility to pneumonia and upper respiratory infections, while exposure to gaseous pollutants causes pulmonary DNA damage and oxidative damage. These findings parallel the conclusion drawn from our data: elevated concentrations of both particulate and gaseous pollutants are associated with the incidence of MP infection in children. The effect of SO_2_ was the most significant, which is consistent with the findings of a study in Ningbo [17]. Additionally, a study in Taiwan [12] reported that, among all the pollutants analyzed, SO_2_ had the strongest correlation with the daily hospitalization rate of children with asthma. Based on this, we can conclude that SO_2_ has the most significant impact on MP infection in children [18]. Our results show that the effect of SO_2_ was most pronounced on the 7th-day lag and the 7th-day cumulative lag. This suggests that the effects of air pollutants on MP infections in children are not immediate but exhibit a certain lag. This finding aligns with previous research, which indicates that air pollution's impact on respiratory diseases often involves both lag and cumulative lag effects [19].

Age-stratified data analysis revealed that children under 6 years of age were more sensitive to SO_2_, whereas children over 6 years of age were more susceptible to NO_2_. A previous study in Suzhou City found that O_3_ was positively correlated with respiratory tract infections in children under 6 years of age [6], a conclusion that differs somewhat from our findings. This discrepancy may be due to differences in study populations, as our research focused exclusively on children with MP infections, excluding those with other respiratory tract infections. The varying sensitivities to SO_2_ and NO_2_ observed across age groups may be explained by “immunization debt,” a term referring to a lack of protective immunity. This phenomenon has contributed to the resurgence of respiratory viruses in some countries since 2021, following the widespread administration of SARS-CoV-2 vaccines and the relaxation of pandemic-related restrictions [20, 21]^.^ Children under 6 years of age have a higher respiratory rate, an immature immune system, and reduced antibody synthesis capacity [22]^.^ Because SO_2_ is an irritant gas, it is more likely to damage their fragile respiratory system, creating favorable conditions for mycoplasma invasion. Lung development in children is a protracted process, commencing in the fetal period and continuing through adolescence [4]. Children under 6 years of age exhibit a higher respiratory rate, narrower airways, and immaturity in both lung development and immune system development. Their capacity for antibody synthesis is reduced, and their antioxidant capacity is weaker. Consequently, their ability to resist oxidative stress associated with air pollution is poorer compared to children over 6 years old. SO_2_, acting as an irritant gas, can more readily damage their vulnerable respiratory system, creating favorable conditions for the invasion of pathogens such as MP. Conversely, the respiratory systems of children over 6 years old are more mature. This maturity provides a more robust foundation for NO_2_ to undergo redox reactions within the respiratory tract. Combined with the relatively low solubility of NO_2_, which allows deeper penetration into the lungs, this makes children over 6 years of age more susceptible to the adverse effects of NO_2_ exposure [5, 23].

Sex-stratified data analysis showed that male children were more sensitive to SO_2_, whereas female children were more sensitive to NO_2_. The underlying reasons for these differences may stem from inherent disparities in airway development between males and females, originating from the early fetal stages and persisting throughout life, or may stem from variations in sex hormone levels. For instance, females possess smaller lungs than males but exhibit a more favorable structural configuration characterized by relatively larger airway diameters relative to lung parenchyma volume [24]. This anatomical feature may contribute to heightened susceptibility to NO_2_ in females. However, research in this specific area remains limited, presenting a promising avenue for future investigation. Furthermore, studies examining air pollution in relation to menopausal hormone levels indicate that increased NO_2_ exposure is significantly associated with altered levels of estradiol and follicle-stimulating hormone during menopause [25]. Conversely, males exhibit greater sensitivity to SO_2_. This may be partially explained by the fact that, while the number of alveoli per unit area and alveolar size show no significant sex differences, boys demonstrate greater lung capacity relative to age and height compared to girls. This likely translates to a larger alveolar surface area in males. Although the age range of subjects in the above studies differs from our selected age group, these findings nonetheless offer relevant theoretical support. Additionally, animal studies suggest that the pulmonary surfactant matures later in males under the influence of androgens compared to that in females [26]. The combination of a larger alveolar surface area and delayed surfactant maturation could potentially afford SO_2_ a greater opportunity for interaction. However, it should be noted that only a limited number of animal experiments have demonstrated that SO_2_ exposure reduces Type II pneumocytes, thereby diminishing the regulatory capacity of the pulmonary surfactant. The results of this study differ from those of He et al. [27], who analyzed the short-term impact and economic burden of air pollutants on children with acute lower respiratory tract infections in Southwest China. Their study concluded that while both boys and girls were susceptible to air pollution, there was no statistically significant difference between the sexes. This inconsistency may be due to variations in research methodologies, study periods, and regional environmental factors.

Seasonally stratified analysis revealed that the impact of air pollutants on MP infections in children was significantly stronger in autumn and winter than in spring and summer. This finding aligns with a time-series on air pollution and the incidence of respiratory and cardiovascular diseases in Beijing [28]. Seasonal variations influence the health effects of air pollution, likely due to factors such as higher concentrations of air pollutants, lower temperatures, and poor air mobility during autumn and winter. A low-temperature environment in autumn and winter may decrease the defense function of children's respiratory mucosa, increasing the risk of infection. Additionally, colder temperatures in these seasons often lead to increased use of motor vehicles, contributing to higher pollutant levels and further exacerbating the risk of MP infection in children.

Our study also found that the cumulative effects of atmospheric pollutants had a significant impact on MP infections in children. This indicates that long-term exposure to air pollutants in children should not be overlooked. While short-term fluctuations in pollutant concentrations may not cause immediate health problems, prolonged exposure to high pollution levels may significantly increase the risk of MP infections in children.

This study had some limitations. First, the data were obtained from a single hospital, which may not fully represent the entire city of Chengdu. Second, individual exposure levels to air pollutants were not considered; instead, it was assumed that exposure levels were uniform across the city. Third, during preliminary experiments with the multipollutant model, we found its results did not significantly differ from those of the single-pollutant model. This may be due to the limited sample size, the relatively narrow scope of the study, and the insufficient duration of the research period in this study. In addition, this study mainly focused on the relationship between air pollutants and MP infection, without considering the influence of other respiratory tract infectious pathogens. Future studies should explore the association between various pathogens and air pollution.

However, this is the first study to investigate the correlation between air pollution and MP infections in children in this region. The findings have significant implications for public health policy. First, our results suggest that increases in atmospheric pollutant concentrations have both lagging and cumulative effects on MP infections in children. Therefore, it is necessary to establish an early warning system for air pollution and children's respiratory tract infection. Second, different protection measures should be implemented for children of different ages, sexes, and seasons. Finally, monitoring and managing atmospheric pollutants should be strengthened to reduce the risk of MP infections in children and protect and promote children's health.

## Figures and Tables

**Table 1:** Summary statistics for air pollutants concentrations and weather conditions in Chengdu, 2019–2023.

	Mean	SD	Percentiles			Max	Min
			25	50	75		
PM_2.5_ (μg/m^3^)	41.81	27.48	22.00	35.00	54.45	188.60	4.40
PM_10_ (μg/m^3^)	65.45	36.76	37.80	57.20	86.40	260.60	7.60
CO (mg/m^3^)	0.71	0.21	0.60	0.69	0.83	2.61	0.38
SO_2_ (μg/m^3^)	5.15	2.10	3.80	5.00	6.40	15.60	0.20
NO_2_ (μg/m^3^)	37.17	15.47	25.00	35.60	47.00	93.20	6.00
O_3_ (μg/m^3^)	94.21	52.54	53.60	82.80	129.05	261.40	3.80
Temperature (°C)	17.05	7.37	10.44	17.38	23.44	32.36	0.75
Humidity (%)	80.00	9.00	74.00	80.50	86.50	99.00	37.00

**Table 2:** Pearson's correlation matrix between air pollutant concentrations and weather conditions in Chengdu, 2019–2023.

	PM_2.5_	PM_10_	CO	SO_2_	O_3_	NO_2_	Temperature	Humidity	Patients
PM_2.5_	1.000	0.953^∗∗^	0.706∗∗	0.454^∗∗^	−0.210^∗∗^	0.714^∗∗^	−0.456^∗∗^	−0.100^∗∗^	−0.073^∗∗^
PM_10_		1.000	0.631^∗∗^	0.468^∗∗^	−0.165^∗∗^	0.756^∗∗^	−0.427^∗∗^	−0.222^∗∗^	−0.061^∗∗^
CO			1.000	0.576^∗∗^	−0.220^∗∗^	0.718^∗∗^	−0.286^∗∗^	0.120^∗∗^	−0.123^∗∗^
SO_2_				1.000	−0.054^∗^	0.619^∗∗^	−0.141^∗∗^	−0.158^∗∗^	−0.277^∗∗^
O_3_					1.000	−0.188^∗∗^	0.768^∗∗^	−0.479^∗∗^	0.007
NO_2_						1.000	−0.310^∗∗^	−0.118^∗∗^	−0.125^∗∗^
Temperature							1.000	−0.096^∗∗^	0.029
Humidity								1.000	0.028
Patients									1.000

^∗^ *p* < 0.05.

^∗∗^ *p* < 0.01.

**Table 3:** Summary statistics of outpatient children with *mycoplasma* infection by sex, age, and season, 2019–2023.

Characteristics	Number of patients	Percentage (%)
Sex		
Boys	9783	46.42
Girls	11,292	53.58
Age (years)		
0–6	15,161	71.94
7–18	5914	28.06
Season		
March–May	4976	23.61
June–August	4251	20.17
September–November	7149	33.92
December–February	4699	22.30
Total	21,075	100

**Table 4:** Summary statistics of children hospitalized for *mycoplasma* infection by sex, age, and season, 2019–2023.

Characteristics	Number of patients	Percentage (%)
Sex		
Boys	3475	49.90
Girls	3489	50.10
Age (years)		
0–6	5162	74.12
7–18	1802	25.88
Season		
March–May	1037	14.89
June–August	1373	19.72
September–November	2583	37.09
December–February	1971	28.30
Total	6964	100

## Data Availability

All data generated or analyzed in this study are included in this article.
